# COVID-19 Preventive Practices among Bus Station Workers in Ethiopia

**DOI:** 10.4269/ajtmh.20-1417

**Published:** 2021-11-05

**Authors:** Mebrahtu Eyasu, Yoseph Worku, Berhan Ababaw, Yifru Berhan

**Affiliations:** ^1^ Saint Paul’s Hospital Millennium Medical College, Department of Pharmacology, Addis Ababa, Ethiopia;; ^2^ Saint Paul’s Hospital Millennium Medical College, Department of Public Health, Addis Ababa, Ethiopia;; ^3^ Saint Paul’s Hospital Millennium Medical College, Department of Biochemistry, Addis Ababa, Ethiopia;; ^4^ Saint Paul’s Hospital Millennium Medical College, Department of Obstetrics and Gynecology, Addis Ababa, Ethiopia

## Abstract

As of May 19, 2021, Ethiopia was among the five African countries most affected by COVID-19. A cross-sectional design was used to assess the level of knowledge, perceptions, and practices of bus station workers about COVID-19 between August 25 and September 17, 2020. Face-to-face interviewer-administered questionnaires were used. To identify the factors associated with the dependent variables, simple and multiple binary logistic regression analyses were used. A *P* value < 0.05 was considered significant. Data were analyzed using SPSS version 20 software. In this study, 427 workers from three bus stations participated. Approximately 84.5%, 84.8%, and 81.3% of the workers had good knowledge, positive perceptions, and good practices, respectively. Multivariable logistic regression analysis showed that workers with a monthly income of 3,001 to 4,000 birr were about four times more likely to have poor knowledge compared with higher income workers. Those workers with poor knowledge were 2.4 times, and security workers were 3.7 times, more likely to have poor practices compared with workers with good knowledge and drivers, respectively. In conclusion, workers used in security and those who had poor knowledge regarding COVID-19 failed to exhibit effective preventative practices against the virus.

## INTRODUCTION

The human coronavirus first reared its ugly head more than five decades ago.^1^ In the past, the virus emerged in the form of Middle East respiratory syndrome and severe acute respiratory syndrome.^2^^,^^3^ The newly identified human coronavirus is named COVID-19, and this outbreak originated in Wuhan City, China, in late December 2019. By April 30, 2020, most countries in the world were suffering the effects of COVID-19, some of which were already burdened by prevailing humanitarian crises.^4^ By May 19, 2021, COVID-19 had spread throughout the entire world, with more than 164 million confirmed cases and more than 3,420,532 deaths attributed to it.^5^

COVID-19 has a high transmission rate with an unclear mechanism, but is spread primarily via respiratory droplets, aerosols, and, to a lesser degree, from contaminated objects.^6^^–^^9^ Disease symptoms include fever, dry cough, fatigue, myalgia, and dyspnea. Severe cases present as an acute respiratory distress syndrome-like picture, with septic shock, intractable metabolic acidosis, and coagulation dysfunction.^10^^,^^11^ An occupation with a high COVID-19 risk of transmission is bus station workers, including bus drivers and cashiers.^12^

The WHO has provided guidance for the prevention and treatment of COVID-19. In addition, countries’ health ministries have also provided guidelines. Adherence to these guidelines may be dependent on the level of knowledge, attitudes, and practices among populations.^13^^–^^16^ In outbreaks, panic or ignorance regarding the spread of infectious diseases may complicate attempts to prevent the spread of the disease.^16^^–^^20^ The rapid spread and mortality of COVID-19 created excessive anxiety among some individuals,^21^ especially those who remained unaffected.^22^

In Africa, the high levels of poverty, poorly developed health systems, and the population density of urban areas portended dire predictions about the virus. Although the jury remains out, it is speculated that a warmer climate, a youthful population, and former experiences of fighting infectious diseases has spared Africa the severest consequences of the pandemic seen on most other continents.^23^

In Addis Ababa, Ethiopia, despite the deaths caused by COVID-19, some inhabitants showed little adherence to the preventive measures promulgated by the government of Ethiopia. COVID-19 could spread at long-distance bus stations, where workers have direct contact with passengers who typically sit in close proximity on long-distance trips (≥ 270 km). We thought an assessment of the level of knowledge, perceptions, and the preventive practices of long-distance bus station workers about COVID-19 might be revealing.

## METHODS

### Study design, period, and setting.

A cross-sectional study design was conducted between August 25 and September 17, 2020, after 4 months of a state of emergency and social distancing. In Ethiopia, between August 25 and September 25, the total number of confirmed cases and deaths increased from 43,688 to 66,913 and from 709 to 1,060, respectively.^24^ Our study was conducted at three long-distance bus stations (in Asko, Autobustera, and Lamberet) in Addis Ababa, Ethiopia. During the pandemic, 600 workers were active at these three stations.

### Participants.

Included in the study were workers 18 years of age or older who managed buses that traveled ≥ 270 km. The minimum required sample size (425 participants) was obtained using the single-population proportion formula, using a 50% proportion and a margin of error of 5%. After the Federal Ministry of Health at St. Paul’s Hospital Millennium Medical College, Research Directorate Office, Addis Ababa, Ethiopia, grant call announcement, an application letter together with our research proposal was submitted by e-mail to the Research Directorate Office of the College for competition. Within 10 days, the assigned grant committee of the Research Directorate Office of the college screened and announced the proposal was one of the grant winner proposals. Furthermore, the directorate notified the institutional review board (IRB) of St. Paul’s Hospital Millennium Medical College of the submission, which submitted to a strict review process. The IRB evaluated and approved the proposal. Subsequently, the study was conducted in the bus stations after obtaining ethical clearance from the IRB and a support letter from the Research Directorate Office. During the data collection period, to decrease the chance of contracting COVID-19 during face-to-face interviews, preventive safety measures against COVID-19 were adhered to. All participants gave their informed consent before participation. For all participants, ethical issues were strictly observed.

### Sampling technique.

In our study, participants were recruited from the three long-distance bus stations using the opportunistic sampling technique. During the COVID-19 crisis, the workers were drivers (*n *= 127), driver supporters (*n *= 57), loaders/uploaders (*n *= 29), cashiers (*n *= 59), guards (gate; *n *= 35), simirits (*n *= 47), security personnel (*n *= 29), cleaners (*n *= 30), and others (daily laborers, gardeners, and administrative staff; * n *= 20).

### Variables, definitions, and outcomes.

The dependent variables were knowledge (good or poor), perception (negative or positive), and practice (good or poor). The questions used to assess the sociodemographic characteristics, knowledge, perception, and practice regarding COVID-19 were the independent variables. Knowledge was the awareness of COVID-19. Perception was the state of preparedness when confronted with COVID-19. Practice was the act of taking preventive measures regarding the virus. Knowledge, perceptions, and practices were measured by calculating the mean score of 15 items (knowledge), seven items (perception), and 11 items (practices). The variables were categorized as good knowledge, positive perceptions, and good practices if participants scored the mean score or more of the correctly answered questions for each category, or as poor knowledge, negative perceptions, and poor practices if participants scored less than the mean score of the correctly answered questions.^25^

The Supplemental questionnaire was modified and adapted from published articles.^15^^,^^26^^–^^29^ Knowledge questions were answered as true or false, or “I don’t know.” The perception and practice questions had only a true or false option. Correct answers were coded as 1 point, incorrect, 0 point. Before analysis, negatively worded items which had correct answers other than the “true” option correct answers were scored reversely. A “simirit” is an individual used at long-distance bus stations whose primary responsibility includes scheduling departure times and setting regional bus routes.

### Data collection and quality management.

Data collectors were trained in the study objectives, data collection methods, quality of data, and communication skills. The pre-structured questionnaire was pretested 2 weeks prior to the actual data collection on 5% of the sample size from Zenebe Work, a long-distance bus station in Addis Ababa. Based on the feedback from the questionnaire, modifications were made. The supervisors’ reviewed data daily for inconsistencies and completeness. All in all, data collection was done through face-to-face interviews.

### Statistical analysis.

Data were coded, entered, cleaned, and analyzed using SPSS version 20 software (SPSS Inc., Chicago, IL). Descriptive analyses (frequency, percentage, and mean) were computed. To determine the level of knowledge, perceptions, and preventive practices of the participants, the means of the corrected answers were calculated. To identify factors associated with the dependent variables, simple and multiple binary logistic regression analyses were conducted. In the simple binary logistic regression, all factors with a *P* value < 0.20 were considered candidates for the multiple binary logistic regression. In all statistical tests, a *P* value < 0.05 was significant.

## RESULTS

### Sociodemographic characteristics.

A total of 427 workers participated in this study. The mean age (±SD) was 35.88 ± 10.80 years (range, 19–78 years). Approximately 127 workers (29.7%) were drivers, 59 (13.8%) were cashiers, and 51 (11.9%) were driver supporters. The workers were predominantly male (*n *= 388, 90.9%) with an age range of 25 to 44 years (*n *= 293, 68.6%), married (*n *= 262, 61.4%), had completed secondary school (*n *= 231, 54.1%), were Orthodox Christians (*n *= 366, 85.7%), and were city residents (*n *= 394, 92.4%). Majority of the workers, 182 (42.6%), were from one of the bus stations (Autobustera) (Table 1).

**Table 1:** Sociodemographic characteristics

Characteristic	*n* (%)
Gender	
Male	388 (90.9)
Female	39 (9.1)
Age group (y)	
18–24	53 (12.4)
25–34	153 (35.8)
35–44	140 (32.8)
45–54	50 (11.7)
≥ 55	31 (7.3)
Education	
Illiterate	11 (2.6)
Read and write	4 (0.9)
Elementary	128 (30)
Secondary	231 (54.1)
College	53 (12.4)
Religion	
Orthodox	366 (85.7)
Muslim	26 (6.1)
Protestant	33 (7.7)
Catholic	1 (0.2)
Other	1 (0.2)
Marital status	
Single	147 (34.4)
Married	262 (61.4)
Divorced	10 (2.3)
Widowed	7 (1.6)
Separated	1 (0.2)
Residency	
Addis Ababa	394 (92.3)
Other*	33 (7.7)
Monthly income (birr)†	
≤ 1,000	73 (17.1)
1,001–2,000	153 (35.8)
2,001–3,000	76 (17.8)
3,001–4,000	35 (8.2)
≥ 4,001	90 (21.1)
Job	
Driver	127 (27.9)
Driver supporter	51 (11.9)
Loader/uploader	29 (6.8)
Cashier	59 (13.8)
Guard (gate)	35 (8.2)
Simirit	47 (11)
Security	29 (6.8)
Cleaner	30 (7)
Other‡	20 (4.7)
Long-distance bus station	
Asko	145 (34)
Autobustera	182 (42.6)
Lamberet	100 (23.4)

y = years.

* Sululta, Gebre Buracha, Keta, Dessie, Gojam, Debrebirhan, and others.

† Conversion of birr to US dollars (USD) August 25, 2020: 1 birr = 0.027746 USD; September 17, 2020: 1 birr = 0.027242 USD.

‡ Daily laborers, gardeners, and administrative staff.

### Long-distance bus station workers’ knowledge.

The workers’ average knowledge score was 11.69 ± 1.38 points (range, 3–15 points). Four hundred thirteen workers (96.7%) were aware of the most common clinical symptoms, 204 (47.8%) could distinguish the virus from the common cold/flu, and 354 (82.9%) believed there was no effective cure. Approximately 383 workers (89.7%) knew that COVID-19 was spread via respiratory droplets of an infected patient. However, 318 workers (74.5%) stated that asymptomatic transmission was possible.

Most workers avoided crowded places (*n *= 396, 92.7%), wore masks (*n *= 399, 93.4%), and understood that contact with an infected person would result in immediate isolation (*n *= 419, 98.1%) with a quarantine time of 14 days (*n *= 410, 96%), and COVID-19 patients would remain in the treatment center until discharge (*n *= 423, 99.1%). About 339 workers (79.4%) reported that COVID-19 was airborne, that eating or touching wild animals would not result in transmission (*n *= 118, 27.6%), that isolation and treatment effectively reduced transmission (*n *= 421, 98.6%), and that children and young adults should take preventive measures (*n *= 347, 81.3%). Overall, good knowledge was demonstrated by 361 workers (84.5%).

### Preventive practices regarding COVID-19.

The mean score for practices was 10 ± 1.20 points. Most workers self-reported that they had not recently visited any crowded places (*n *= 306, 71.7%); that they wore face masks (*n *= 413, 96.7%); that they stopped shaking hands (*n *= 416, 97.4%); that they frequently washed their hands with water and soap (*n *= 421, 98.6%); that they avoided touching their eyes, nose, and mouth before handwashing (*n *= 407, 95.3%); that they discarded used masks in dust bins (*n *= 414, 97%), and that they followed government instructions (*n *= 420, 98.4%). After work, however, 95 workers (22.2%) did not stay home, 52 (12.2%) did not avoid close proximity (within 2 m), and 26 (6.1%)—during coughing and/or sneezing—did not use a tissue or cough into their elbow. The majority of the workers (*n *= 347, 81.3%) demonstrated good practices.

### Long-distance bus station workers’ perceptions.

The mean perception score was 5.5 ± 1.07 points. Most of the workers received health education about COVID-19 (*n *= 361, 84.5%), agreed that COVID-19 would be controlled successfully (*n *= 360, 84.3%), had confidence that Ethiopia could win the battle against COVID-19 (*n *= 389, 91.1%), believed the Ethiopian government handled the crisis very well (*n *= 376, 88.1%), and thought that COVID-19-infected individuals might be stigmatized (*n *= 270, 63.2%).

In response to statements posed to the participants, the following self-reported results were found: 127 workers (29.7%) reported they would get infected despite practicing the safety measures, 157 workers (36.8%) believed COVID-19 patients would be stigmatized by those who knew their health status, and, if infected, most workers (*n *= 412, 96.5%) thought there were risks for themselves and their families. Most of the workers had positive perceptions (*n *= 362, 84.8%).

### Factors associated with knowledge, practices, and perceptions.

Multivariable logistic regression analysis showed that workers with a monthly income of 3,001 to 4,000 birr were about four times more likely to have poor knowledge compared with higher income workers (adjusted odds ratio [AOR], 3.929; 95% CI, 1.326–11.640). Married workers were 47.3% less likely to have poor COVID-19 knowledge compared with single workers (AOR, 0.527; 95% CI, 0.286–0.972) (Table 2).

**Table 2:** Multivariable logistic regression of workers’ knowledge about COVID-19 in Addis Ababa, Ethiopia

Predictor	Knowledge level		COR (95% CI), *P* value	AOR (95%, CI), *P* value
	Poor, n (%)	Good, n (%)		
Marital status				
Single	34 (23.1)	113 (76.9)	1	1
Married	31 (11.8)	231 (88.2)	0.446 (0.261–0.762), 0.003	0.527 (0.286 – 0.972), 0.040
Others*	1 (5.6)	17 (94.4)	0.196 (0.025–1.523)	0.227 (0.027 – 1.876)
Residency				
Addis Ababa	58 (14.7)	336 (85.3)	1	1
Other	8 (24.2)	25 (75.8)	1.854 (0.797–4.309)	2.451 (0.983 – 6.112), 0.055
Monthly income (birr)				
≥ 4,001	8 (8.9)	82 (91.1)	1	1
≤ 1,000	11 (15.1)	62 (84.9)	1.819 (0.690–4.791)	0.769 (0.166 – 3.559)
1,001–2,000	28 (18.3)	125 (81.7)	2.296 (0.998–5.285)	1.345 (0.342 – 5.297)
2,001–3,000	9 (11.8)	67 (88.2)	1.377 (0.504–3.764)	1.034 (0.278 – 3.843)
3,001–4,000	10 (28.6)	25 (71.4)	4.100 (1.461–11.506), 0.007	3.929 (1.326 – 11.640), 0.014
Job				
Driver	17 (13.4)	110 (86.6)	1	1
Driver supporter	9 (17.6)	42 (82.4)	1.387 (0.574–3.352)	1.371 (0.338–5.568)
Loader/uploader	3 (10.3)	26 (89.7)	0.747 (0.204–2.739)	1.056 (0.219–5.088)
Cashier	16 (27.1)	43 (72.9)	2.408 (1.117–5.191), 0.025	2.374 (0.642–8.783)
Guard (gate)	8 (22.9)	27 (77.1)	1.917 (0.749–4.907)	2.782 (0.633–12.219)
Simirit	5 (10.6)	42 (89.4)	0.770 (0.267–2.220)	0.666 (0.192–2.311)
Security	4 (13.8)	25 (86.2)	1.035 (0.3203.344)	1.501 (0.329–6.844)
Cleaner	3 (10)	27 (90)	0.719 (0.196–2.632)	0.807 (0.148–4.414)
Other	1 (5)	19 (95)	0.341 (0.043–2.712)	0.479 (0.053–4.311)

AOR = adjusted odds ratio; COR = crude odds ratio; CI = confidence interval; n (%) is number (percentage).

* Divorced, Widowed, and Separated.

Workers with poor knowledge were 2.4 times more likely to have poor practices compared with workers with good knowledge (AOR, 2.383; 95% CI, 1.252–4.538), and security workers were 3.7 times more likely to have poor practices compared with drivers (AOR, 3.721; 95% CI, 1.098–12.609). In addition, workers with secondary-level education were 59.2% less likely to have poor COVID-19 practices compared with workers with college-level education (AOR, 0.408; 95% CI, 0.187–0.891) (Table 3).

**Table 3:** Multivariable logistic regression of workers’ preventive practices for COVID-19 in Addis Ababa, Ethiopia

Predictor	Practice level		COR (95% CI), *P* value	AOR (95% CI), *P* value
	Poor, n (%)	Good, n (%)		
Education				
College	19 (35.8)	34 (64.2)	1	1
Informal	1 (6.7)	14 (93.3)	0.128 (0.016– 1.049)	0.194 (0.020–1.892)
Elementary	26 (20.3)	102 (79.7)	0.456 (0.225–0.925), 0.030	0.554 (0.237–1.291)
Secondary	34 (14.7)	197 (85.3)	0.309 (0.158–0.603), 0.001	0.408 (0.187–0.891), 0.024
Monthly income				
≥ 4,001	13 (14.4)	77 (85.6)	1	1
≤ 1,000	11 (15.1)	62 (84.9)	1.051 (0.440– 2.508)	0.639 (0.176–2.318)
1,001–2,000	28 (18.3)	125 (81.7)	1.327 (0.648–2.716)	0.828 (0.268–2.560)
2,001–3,000	21 (27.6)	55 (72.4)	2.262 (1.044–4.901), 0.039	1.376 (0.489–3.873)
3,001–4,000	7 (20)	28 (80)	1.481 (0.536–4.088)	1.125 (0.377–3.359)
Knowledge level				
Good	60 (16.6)	301 (83.4)	1	1
Poor	20 (30.3)	46 (69.7)	2.181 (1.205–3.949), 0.01	2.383 (1.252–4.538), 0.008
Job				
Driver	16 (12.6)	111 (87.4)	1	1
Driver supporter	11 (21.6)	40 (78.4)	1.908 (0.817–4.457)	2.419 (0.714–8.203)
Loader/uploader	6 (20.7)	23 (79.3)	1.810 (0.640–5.121)	2.002 (0.558–7.186)
Cashier	9 (15.3)	50 (84.7)	1.249 (0.517–3.017)	1.299 (0.383–4.412)
Guard (gate)	6 (17.1)	29 (82.9)	1.435 (0.516–3.994)	1.585 (0.385–6.521)
Simirit	12 (25.5)	35 (74.5)	2.379 (1.027–5.506), 0.043	1.721 (0.578–5.125)
Security	11 (37.9)	18 (62.1)	4.240 (1.698–10.586), 0.002	3.721 (1.098–12.609), 0.035
Cleaner	3 (10)	27 (90)	0.771 (0.209–2.837)	1.166 (0.225–6.054)
Other	6 (30)	14 (70)	2.973 (0.999–8.848), 0.05	2.825 (0.757–10.546)

AOR = adjusted odds ratio; COR = crude odds ratio; CI = confidence interval; n (%) is number (percentage).

Workers with elementary and secondary education were 64.4% (AOR, 0.356; 95%, CI, 0.141–0.899) and 57.0% (AOR, 0.430; 95% CI, 0.194–0.954) less likely to have poor perceptions compared with those with college-level education, respectively (Table 4).

**Table 4:** Multivariable logistic regression of workers’ perception about COVID-19 in Addis Ababa, Ethiopia

Predictor	Perception level		COR (95% CI), *P* value	AOR (95% CI), *P* value
	Negative, n (%)	Positive, n (%)		
Education				
College	14 (26.4)	39 (73.6)	1	1
Informal	2 (13.3)	13 (86.7)	0.429 (0.086–2.142)	0.362 (0.059–2.212)
Elementary	15 (11.7)	113 (88.3)	0.370 (0.164–0.835), 0.017	0.356 (0.141–0.899), 0.029
Secondary	34 (14.7)	197 (85.3)	0.481 (0.236–0.979), 0.043	0.430 (0.194–0.954), 0.038
Marital status				
Single	30 (20.4)	117 (79.6)	1	1
Married	33 (12.6)	229 (87.4)	0.562 (0.327–0.967), 0.037	0.598 (0.322–1.109)
Other	2 (11.1)	16 (88.9)	0.488 (0.106–2.237)	0.610 (0.127–2.934)
Job				
Driver	15 (11.8)	112 (88.2)	1	1
Driver supporter	6 (11.8)	45 (88.2)	0.996 (0.363–2.728)	0.853 (0.296–2.459)
Loader/uploader	6 (20.7)	23 (79.3)	1.948 (0.683–5.553)	2.095 (0.717–6.125)
Cashier	14 (23.7)	45 (76.3)	2.323 (1.037–5.203), 0.040	1.836 (0.776–4.348)
Guard (gate)	6 (17.1)	29 (82.9)	1.545 (0.551–4.332)	1.346 (0.451–4.013)
Simirit	9 (19.1)	38 (80.9)	1.768 (0.716–4.370)	1.069 (0.395–2.889)
Security	4 (13.8)	25 (86.2)	1.195 (0.365–3.908)	1.094 (0.321–3.732)
Cleaner	4 (13.3)	26 (86.7)	1.149 (0.352–3.748)	1.311 (0.353–4.874)
Other	1 (5)	19 (95)	0.393 (0.049–3.151)	0.275 (0.032–2.323)

AOR = adjusted odds ratio; COR = crude odds ratio; CI = confidence interval; n (%) is number (percentage).

## DISCUSSION

In this study, multiple binary logistic regression analyses showed that workers with secondary education had a significant association with poor practices. This finding is similar to studies in Ethiopia^30^ and India.^31^ A study in Iran^32^ and Pakistan^33^ concluded that a higher level of education was associated with high preventive practices. In general, education is one of the contributing factors that affects healthy actions.^34^ However, those with lower education might have a challenge in seeking information on how to practice preventive safety measures against COVID-19 infection. Therefore, because of their lower educational status, workers might have poor practices with regard to COVID-19 prevention.^35^

There was a strong and significant association between working as security personnel and poor practices. This is supported by the results of a study conducted in Uganda.^27^ In our study, more than 80% of all workers exhibited good practices, which is similar to the findings in Uganda^27^ and Vietnam.^36^ Workers with poor knowledge about COVID-19 were significantly associated with poor practices. This finding is consistent with reports from Ethiopia^30^ and Vietnam.^36^ Considering such practices, the investigators recommended that these populations be targeted for teaching about safety measures and how to apply them.^2^^,^^6^ Furthermore, this finding is in line with the association of good knowledge about COVID-19.^27^

Workers in our study with monthly incomes between 3,001 and 4,000 birr were significantly associated with poor knowledge compared with those with higher incomes. This result is similar to other studies.^30^^,^^37^^,^^38^ Higher income participants were associated with better understanding and safer practices.^15^ Economic status appeared to be a central factor with regard to maintaining recommended health practices.^39^

Married workers were also less likely to have poor knowledge about COVID-19 compared with those workers who were single. This finding is similar to an earlier report from Ethiopia.^40^ However, a previous study found that^41^ unmarried people were more likely to have good knowledge of COVID-19.

Our study revealed that a large number of workers had positive perceptions about COVID-19, similar to a report from Nigeria,^42^ and more than those found in a study from Uganda.^27^ Although there is no published evidence for comparing this finding, workers with elementary and secondary education were significantly associated and less likely to have a poor perception of COVID-19. In our study, nearly 85% of workers had good knowledge. Studies in Tanzania^28^ and Uganda^27^ reported similar findings.

Most of the workers in our study had prior health education. More than 90% knew the main clinical symptoms of COVID-19, similar to findings in a study from Nepal,^43^ but more than the number found a study from Ethiopia.^26^ A large number of workers in our study felt confident that Ethiopia could win the battle against COVID-19. This sentiment matches the results of previous studies.^15^^,^^28^^,^^37^

Nearly three quarters of the workers in our study knew that older people and/or people with chronic illnesses were at greater risk of developing a severe form of COVID-19. This has been supported by previous studies.^26^^,^^29^^,^^44^^,^^45^ A small portion of the workers still perceived that COVID-19 infected only the elderly, which is similar to reports from Nepal and Pakistan.^43^^,^^46^

Our study also revealed that almost three quarters of workers knew about asymptomatic transmission of COVID-19, which is inconsistent with previous studies in Ethiopia^26^ and Saudi Arabia.^47^ This discrepancy might be a result of the limited sample size and the length of time since the outbreak of the virus. More than half the workers understood the reason for social distancing and the time periods of quarantine, treatment, and discharge. These findings are similar to previous studies.^43^^,^^47^^,^^48^

Almost all the workers in our study self-reported to wearing masks. This report is consistent with a study conducted in China.^15^ Earlier studies, however, revealed less mask adherence.^36^^,^^37^^,^^42^^,^^46^^,^^49^^–^^51^ Reasons could be participants’ beliefs about COVID-19 prevalence, the amount of time since the outbreak, or sporadic public service education. Also, changing guidelines disseminated by the WHO^52^ and the Centers for Disease Control and Prevention could be influencing factors.^53^

A large number of workers self-reported coughing and/or sneezing into their elbows/masks. In addition, they did not touch their eyes, nose, or mouth before handwashing or hand sanitizing. These findings are similar to those from Ethiopia,^40^ the Philippines,^54^ Pakistan,^50^ and Nigeria.^42^ In the Indian study, compliance was found to be less.^49^

One study limitation included workers’ attempts to give socially acceptable, correct responses to be more accurate—particularly for the perception and practice questions.^55^ To mitigate this, data collectors stressed that actual status reflecting responses was critical.

In conclusion, clearly, some workers exhibited poor COVID-19 preventative practices. Because the nature of their work engenders frequent and close proximity to the general public, the impact of this failure cannot be underestimated. Reformatting and redirecting training protocols to address the personal and/or emotional perspectives of these workers would likely result in better compliance. Presentations must be delivered by a knowledgeable, engaged, and enthusiastic training team. Last, a schedule of monitoring must be initiated to verify the program’s validity and/or deficiencies accurately.

## Supplemental tables


Supplemental materials
