# Supplementary material for: COVID-19 Preventive Practices among Bus Station Workers in Ethiopia
Source: Am J Trop Med Hyg. 2021 Nov 5;106(1):114–20. doi: 10.4269/ajtmh.20-1417 (PMC8733506; doi:10.4269/ajtmh.20-1417)
Supplement: Supplementary file 1 [file tpmd201417.SD1.pdf]

Data Collection Questionnaire (English)

Instructions: Write your response on the space provided for open ended questions and Encircle your Response for close ended questions.

Part-1: Sociodemographic variables

QN.	Question	Response
1	Age	_____________
2	Sex	A. Male B. Female
3	Religion	A. Orthodox<br>B. Muslim<br>C. Protestant<br>D. Catholic<br>E. Other____
4	Educational status	A. unable to read and write<br>B. Able to read and write<br>C. Primary /1-8th grade/<br>D. Secondary /9-12th grade/<br>E. College and above
5	Marital status	A. Single<br>B. Married<br>C. Divorced<br>D. Widowed<br>E. Separated
6	Occupation	A. Bus driver<br>B. Daily laborer<br>C. Facilitator of the station<br>D. Driver supporter<br>E. Other _____
7	Monthly Income (birr)	_____________________

Part-2: Participant knowledge of COVID-19.

QN.	Knowledge Questions	True	False	I don’t know
Q1	The main clinical symptoms of COVID-19 are fever, fatigue, dry cough, and body aches.			
Q2	Unlike the common cold, stuffy nose, runny nose, and sneezing are less common in persons infected with the COVID-19 virus.			
Q3	There currently is no effective cure for COVID-19, but early symptomatic and supportive treatment can help most patients recover from the infection			
Q4	Not all persons with COVID-2019 will develop to severe cases. Only those who are elderly and have chronic illnesses are more likely to be severe cases			
Q5	Eating or touching wild animals would result in the infection by the COVID-19 virus.			
Q6	Persons with COVID-19 cannot infect the virus to others if they do not have a fever.			
Q7	The COVID-19 virus spreads via respiratory droplets of infected individuals			
Q8	The COVID-19 virus is airborne.			
Q9	Ordinary residents can wear face masks to prevent the infection by the COVID-19 virus.			
Q10	It is not necessary for children and young adults to take measures to prevent the infection by the COVID-19 virus.			
Q11	To prevent the infection by COVID-19, individuals should avoid going to crowded places and avoid taking public transportations.			
Q12	Isolation and treatment of people who are infected with the COVID-19 virus are effective ways to reduce the spread of the virus.			
Q13	People who have contact with someone infected with the COVID-19 virus should be immediately isolated in a proper place.			
Q14	People who have contact with someone infected with the COVID-19 virus should be immediately isolated in a proper place for the isolation period of 14 days.			
Q15	People who have infected with the COVID-19 virus will stay in the isolated COVID-19 treatment center until the patient is negative for the COVID-19 test and approved by health Workers and permit the individual discharged.			

QN.	Practice Questions	Yes	No
Q 1	Over the last few days, have you gone to any crowded place?		
Q 2	Over the last few days, have you worn a mask when leaving home?		
Q 3	Over the last few days, did you stop shaking hands of other people for greeting?		
Q 4	Over the last few days, frequently washing hands with water and soap		
Q 5	Over the last few days, did you avoid close proximity including while greeting (within 2 meter)?		
Q 6	Over the last few days, did you avoid touching eye, nose, mouth before washing hands?		
Q 7	Over the last few days, did you use cover/elbow for coughing/sneezing?		
Q 8	Over the last few days, have you started to stay home?		
Q 9	Over the last few days, did you use hand sanitizer/alcohol?		
Q 10	Over the last few days, did you discard appropriately the used mask in dust bin?		
Q 11	Generally, do you follow the COVID-19 prevention measures delivered by the government?		

Part-3: Practices of participants on COVID-19.

Part-4: Participant perception of COVID-19.

QN.	Perception Questions	Yes	No
Q 1	Did you get health education about COVID-19?		
Q 2	Do you agree that COVID-19 will be successfully controlled?		
Q 3	Do you have the confidence that Ethiopia can win the battle against the COVID-19 virus?		
Q 4	The government of Ethiopia is handling the COVID-19 health crisis very well.		
Q 5	Do you think COVID-19 infection leads to stigma on those people who have COVID-19 infection?		
Q 6	Do you think that If I am infected, it has risks to me and my family?		
Q 7	Although I used to practice the safety prevention measures, it is must to acquire the COVID-19 infection.		
